# Stable between‐subject statistical inference from unstable within‐subject functional connectivity estimates

**DOI:** 10.1002/hbm.24442

**Published:** 2018-10-25

**Authors:** Diego Vidaurre, Mark W. Woolrich, Anderson M. Winkler, Theodoros Karapanagiotidis, Jonathan Smallwood, Thomas E. Nichols

**Affiliations:** ^1^ Wellcome Trust Centre for Integrative Neuroimaging Oxford Centre for Human Brain Activity, University of Oxford Oxford UK; ^2^ Emotion and Development Branch National Institute of Mental Health, National Institutes of Health Bethesda Maryland; ^3^ Department of Psychiatry Yale University School of Medicine New Haven Connecticut; ^4^ Department of Psychology University of York York UK; ^5^ Big Data Institute University of Oxford Oxford UK

**Keywords:** dynamic functional connectivity, functional connectivity, hidden Markov model, hypothesis testing, multiple replications, permutation testing, statistical testing, test combination

## Abstract

Spatial or temporal aspects of neural organization are known to be important indices of how cognition is organized. However, measurements and estimations are often noisy and many of the algorithms used are probabilistic, which in combination have been argued to limit studies exploring the neural basis of specific aspects of cognition. Focusing on static and dynamic functional connectivity estimations, we propose to leverage this variability to improve statistical efficiency in relating these estimations to behavior. To achieve this goal, we use a procedure based on permutation testing that provides a way of combining the results from many individual tests that refer to the same hypothesis. This is needed when testing a measure whose value is obtained from a noisy process, which can be repeated multiple times, referred to as replications. Focusing on functional connectivity, this noisy process can be: (a) computational, for example, when using an approximate inference algorithm for which different runs can produce different results or (b) observational, if we have the capacity to acquire data multiple times, and the different acquired data sets can be considered noisy examples of some underlying truth. In both cases, we are not interested in the individual replications but on the unobserved process generating each replication. In this note, we show how results can be combined instead of choosing just one of the estimated models. Using both simulations and real data, we show the benefits of this approach in practice.

## 1 INTRODUCTION

Suppose that we are interested in testing hypotheses about variables, or set of variables, which we can observe on multiple occasions such that we may obtain a number of noisy measures of the same underlying (unobserved) feature or process. This can happen when we replicate a measurement on multiple occasions for each subject, or if the design of the experiment is such that the repetitions are independent of each other (which would not be the case, for example, if there is a strong effect of learning or habituation across runs). This can also happen when we are modeling data using an approach that is complex enough that inferences about the model parameters can be slightly different every time we estimate the model, for example, with different arbitrary initializations. This is the case, for example, for independent component analysis (ICA, Hyvarinen & Oja, 2000; Beckmann, DeLuca, Devlin, & Smith, 2005) and Hidden Markov models (HMM, Rabiner, 1989; Vidaurre et al., 2016).

In nondeterministic approaches such as ICA and HMM, the degree to which different initializations will lead to different estimates (i.e., different local minima) of the model parameters depends on elements such as the signal‐to‐noise ratio, training parameters, and amount of available data (Himberg, Hyvärinen, & Exposito, 2004). Successive runs of the algorithm may find local minima that are equally good or equally likely, or it may find suboptimal local minima. While in some settings an appropriate figure of merit (e.g., residual sum of squares or model evidence) can adjudicate between these different estimates, sometimes no practical or definitive model comparison score is available; furthermore, even when a score is available, this is typically an approximation or a heuristic, and it is possible that many models with very similar scores will be found. Here we claim that all models are potentially useful and that an effective combination can be more powerful than choosing a single model. More specifically, in this work, we take up the issue of making inference on these noisy replicate estimates, relating the estimates on a group of subjects to variables such as demographics, behavior or personality scores. For this, we are not interested in whether each score relates to each individual replicate; rather, we aim to assess, based on a single global test over the pool of estimates, whether there is evidence that each score holds a significant association with the estimated measure.

Based on the principles of permutation testing, this article presents a simple approach where we use the *non‐parametric combination* NPC algorithm (Pesarin & Salmaso, 2010; Winkler et al., 2016) to combine results from multiple functional connectivity (FC) estimations, regardless of whether the replications are at the level of data acquisition or model inference. This approach is useful in estimating effects that explain the underlying data that is the focus of the analysis. We demonstrate the validity of this method on the HMM, using simulations and data from the Human Connectome Project (Smith et al., 2013), where we test a measure of (resting state fMRI) dynamic FC over 100 different HMM runs against a number of behavioral variables measured across hundreds of subjects.

## 2 METHODS

### 2.1 Background

We refer to the noisy samples or parameter inference runs as *R replications*, to be distinguished from the *P observed variables* against which we aim to test. (Replications are not to be confused with *realizations*, which we will use to refer to the multiple instances of the synthetic experimental scenario carried out below). We have one hypothesis per observed variable and wish to combine the tests across multiple replications, with no particular interest in assessing each replication in isolation. For *N* subjects, let us denote replications as ***Y*** (*N* by *R*), and observed variables as ***X*** (*N* by *P*). For reference, we will consider each column of ***Y*** (referred to as ***y***_j_) as a noisy sample of the certain unobservable variable of interest *Y*_0_.

For each column of ***Y*** and each column of ***X*** (referred to as ***x***_i_), we can use permutations (Nichols & Holmes, 2002) to test the null hypothesis that there is no association between the model and the observed data. From this procedure, we obtain a (1 by *R*) vector of *p* values per observed variable, say ***p***_j_. A simple approach could combine these *R* values with a simple statistic such as the mean or the median of ***p***_j_ to assess the significance: if the mean *p* value is small (e.g., below 0.01), this would suggest that there is a significant relationship between *Y*_0_ and ***x***_j_. In what follows, we will refer to this summarised *p* value as *p*_mean_, similar to Edgington's *p* value combining method comprised of the sum of *p* values (Edgington, 1972). A more effective approach is to use the geometric mean, equivalent to exponentiating the average of the log *p* values; this is related to Fisher's *p* value combining method (Fisher, 1932) and amplifies the importance of values near zero. Denoting the individual *p*values for a given observed variable of interest as *p*_i_, we have

(1) $$p_{\text{gmean}}=\exp\;\left(\Sigma_{i}\log\left(p_{i}\right)/R\;\right).$$

Again, if *p*_gmean_ is below a certain level, we can state there is a significant relationship between the replications and the examined observed variable. Note that neither *p*_mean_ or *p*_gmean_ are *p* values because they do not distribute uniformly in [0,1] under the null.

### 2.2 Example case for a single pair of variables

Before coming to a complete description we consider a toy example to make the point above more concrete. We wish to assess if there is a linear relationship between two variables, ***a*** and ***b***. The first one, ***a***, with values *a*_n_, is Gaussian distributed (mean 0, standard deviation [*SD*] 1); the second one, ***b***, is a corrupted version of ***a*** by the introduction of random noise:

$$b_{n}=\kappa \;a_{n}+\varepsilon_{n},\;\text{for}\;n=1\ldots N,$$

where *ε*_n_ are independent, Gaussian distributed random variables (mean 0, *SD* = 1), and *κ* ≥ 0**.** We generate replicates of ***b*** based on independent realizations of noise *ε*_n_ and *κ*, where *κ* is randomly sampled from a uniform distribution between 0 and *c.* We choose *c* to define the expected strength of the relationship between ***a*** and ***b***. We then run permutation testing on each data set. We evaluate the power of the permutation combining method to detect a relationship between ***a*** and ***b*** for different values of *c* > 0. Even when *κ* is randomly small on some replicates, it may be large on others (allowing to detect the underlying relationship in these cases).

**Figure 1:**
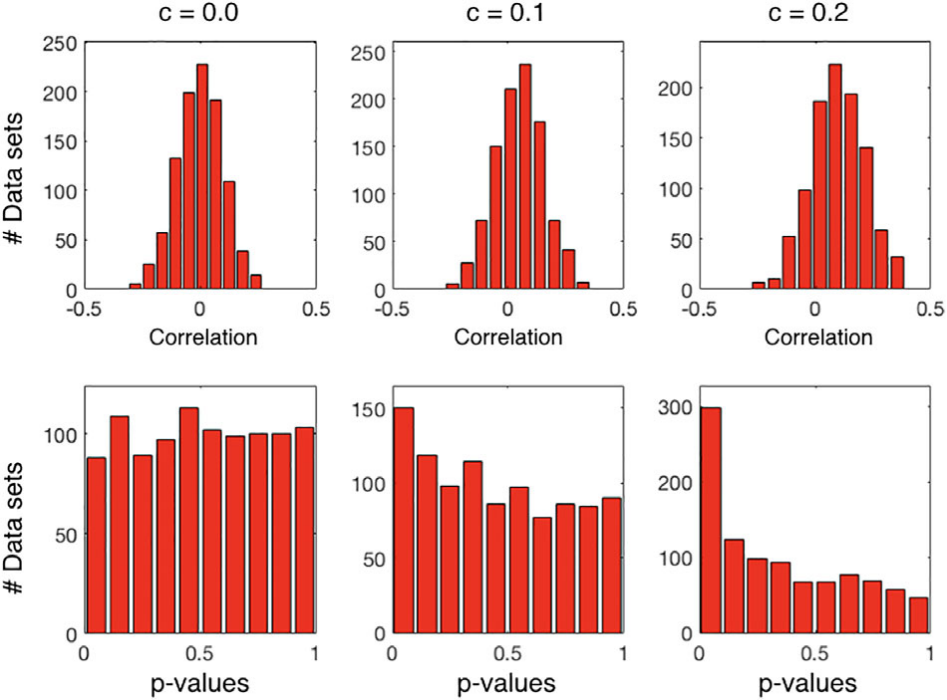
Distribution of correlation and (first‐level) *p* values for the toy example. Simulated examples where we generated 1,000 data sets, where maximum regression coefficient, *c*, is systematically varied. When *c* > 0.0, the mean correlation across data sets is higher than zero (top), and the distribution of *p* values is skewed toward 0.0 (bottom). However, both *p*_mean_ and *p*_gmean_ are higher than .05 [Color figure can be viewed at http://wileyonlinelibrary.com]

For the purpose of illustration, we generated 1,000 data sets using *N* = 100, each with a different value of *κ* sampled from a uniform distribution and performed permutation testing for each of them. We repeat this for three different values of *c*: 0.0, 0.1, and 0.2. Figure 1 shows histograms of correlation coefficients between ***a*** and ***b*** across data sets (top), and histograms of *p* values (bottom). If the empirical distribution of *p* values is basically flat, as is the case when *c* = 0.0, then there is no evidence of a relationship between ***a*** and ***b**.* However, when *c* = 0.1 or *c* = 0.2, then the distribution of *p* values gets increasingly skewed toward zero despite the generally low correlations. Therefore, if ***a*** and ***b*** were experimental replications of some pair of unobserved processes, we could intuitively say that there are signs of correlation between these processes in the *c* = 0.1 and *c* = 0.2 cases. However, neither *p*_mean_ or *p*_gmean_ (data not shown in the figure) are below 0.05; they are higher than 0.2 in all cases, emphasizing again the point that *p*_mean_ or *p*_gmean_ are not *p* values and, thus, the need for a permutation procedure to learn their null distribution.

### 2.3 The NPC algorithm

Given a data set with *N* subjects, we are interested in the relationship between the underlying variables (of which the replications are noisy observations), and not on the individual replications. Since *p*_mean_ or *p*_gmean_ cannot be interpreted as *p* values, we require a method to estimate actual *p* values, that is, distributed uniformly under the null hypothesis. For this, we use the NPC algorithm on *p*_gmean_ (Pesarin & Salmaso, 2010; Winkler et al., 2016). In the case when there is only one variable in the model (*p* = 1), referred to as ***x***, NPC (on *p*_gmean_) proceeds as follows:

- Run statistical tests (e.g., *t* tests) between each replication ***y***_*j*_ and ***x*** to obtain an (*R* by 1) vector of *p* values ***p*** ^0^. We summarise ***p*** ^0^ using the geometric mean, which, using Equation (1), yields *p*_gmean_. This corresponds to the first‐level permutation testing.
- Under the null hypothesis that each replication ***y***_j_ and ***x*** are not associated, we randomly permute ***x*** a number of times *K.* For each permutation *k*, we produce an (*R* by 1) vector of parametric *p* values ***p*** ^k^ analogously to the previous step. We summarise ***p*** ^k^ using the geometric mean, obtaining a surrogate *p* value *p* ^k^_gmean_ per permutation.
- At the second level, we obtain a final value by computing the proportion of surrogate *p* values *p* ^k^_gmean_ that are equal to or lower than the unpermuted summary *p* value *p*_gmean_:

(2) $$p_{\mathrm{NPC}}=\left({\#}_{k}\left\{p_{\text{gmean}}\geq {p^{k}}_{\text{gmean}}\right\}+1\right)/\left(K+1\right).$$

For the *p* > 1 case, that is, when there is more than one observed variable of interest, this procedure can be repeated for each variable, using Equation (1) on the ***x***_i_ separately. Crucially, we would use the same exact same permutations—that is, with the permutations happening in synchrony for all observed variables. This way, the dependence between the tests across variables is implicitly accounted for; in Winkler et al. (2016), this is referred to as “multiple models”. This will yield a final *p* value per observed variable, say *p*_NPC,j_. We can obtain a summary, family‐wise error corrected *p* value (Nichols & Hayasaka, 2003) for each variable of interest *j* by computing

(3) $${p^{\mathrm{FWE}}}_{\mathrm{NPC},j}=\left({\#}_{k}\left\{p_{\text{gmean},j}\geq \min_{j}\left({p^{k}}_{\text{gmean},j}\right)\right\}+1\right)/\left(K+1\right),$$

where *p* ^k^_gmean,j_ is the null surrogate *p* value obtained with Equation (1) for the *j* ^th^ variable of interest and *k* ^th^ permutation. Alternatively, we can use false‐discovery rate (FDR; Benjamini & Hochberg, 1995; Nichols & Hayasaka, 2003) on the uncorrected *p* values *p*_NPC,j_ to obtain FDR‐corrected *p* values *p* ^FDR^_NPC,j_.

In summary, this procedure draws statistical power from both working in logarithmic space (i.e., promoting the importance of *p* values closer to zero), and simultaneously relaxing the alternative hypothesis from the highly conservative “*all* of the replications bear a relationship with the corresponding observed variable” to the less conservative “*at least* some of the replications bear a relationship with the corresponding observed variable”. In the above example, for instance, this scheme of permutation testing produced a *p* value higher than 0.5 when *c* = 0.0, and *p* values lower than 0.001 for both the *c* = 0.1 and *c* = 0.2 cases, exhibiting both sensitivity and robustness to nonnormality (given that no distributional assumptions are made).

MATLAB scripts for the NPC algorithm and the simulations below can be found in Github.^1^

### 2.4 Regression‐based permutation testing

For comparison with the NPC, we briefly outline here an alternative also based on the principles of permutation testing, but where we use multivariate regression in order to integrate over replications. That is, instead of performing univariate statistical testing between each replication and each behavioral variable and then combining the resulting first‐level *p* values using the geometric mean (Step I in the NPC algorithm outlined above), now we use multivariate regression where we predict each behavioral variable using all replications as predictors; we used regularised ridge regression (using a minimal penalty) to alleviate overfitting in the regression and to avoid algebraic indeterminacies when *R* > *N.* Instead of a *p* value combining function with NPC, an *F*‐test is used to summarise all the regression coefficients (i.e., to integrate across replications), and this F score is converted to a *p* value parametrically. We embed this estimation into a standard permutation testing procedure. The final *p* value is eventually computed as in Step III. We shall refer to it as *p*_regr_.

## 3 SIMULATIONS

To illustrate the power of combining FC estimations using NPC, we simulated synthetic data sets emulating a scenario in which we are interested in testing whether FC between a pair of brain regions holds a relation to certain behavioral trait in a set of *N* subjects. In this situation, we have the following variables:

- A subject‐specific FC coefficient *β*, which we cannot observe directly.
- A behavioral variable hypothesized to be related to FC and encoded by a (*N* by 1) vector ***x*,** that can be observed directly.
- Some neural process modulated by *β* denoted as ***S***, which we cannot observe directly. We can consider ***S*** to be some archetypical, noiseless brain activity controlled by *β.*
- The observed (e.g., neuroimaging) data sets ***D***, which are noisy measurements of ***S*** and have a dimension (*T* by 2). This measurement can be repeated up to *R* times per subject.
- An (*N* by *R*) matrix ***Y***, such that *Y*_*nj*_ contains the estimated FC value for the *n* ^th^ subject and *j* ^th^ experimental replication (i.e., the correlation coefficient between the channels of the corresponding measured data ***D***).

**Figure 2:**
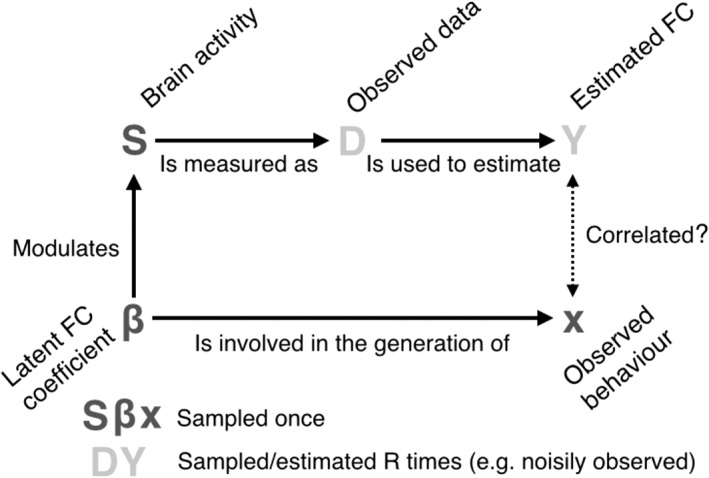
Schematic of the model used for the simulations analysis. The dotted arrow represents the correlation we are testing

A schematic of this experimental case is presented in Figure 2 for clarity. As explained in detail below, the value of *β* is specific for each subject, and its mean over subjects is zero by design. The hypothesis that we are here testing, therefore, is not whether *β* is different from zero, but whether there is an association between *β* and behavior (represented by ***x***). The objective of this simulation is then to assess whether the proposed approach can uncover such relationship, mirroring real data situations often found in the literature where the interest is relating functional connectivity to subject phenotypes (e.g., Smith et al., 2015). Note that, regardless of the generating model for ***Y***, the final goal is to test the relation between ***x*** and ***Y***, and the NPC algorithm could have been applied similarly to other generative models.

We next provide details about the generating process for ***x*** and ***Y***. In this specific context, the noise in the observations (or replications) stems from the imperfect measurement of ***S***, which we can measure multiple times (*R*). Therefore, there is a relation between FC (*β*, which we cannot observe but we can estimate) and behavior (***x***), but this relationship is noisy and weak for some replications. In detail, we generated data from this setting as follows.

We have *N* = 200 subjects. We uniformly sampled a value *β*_n_ between −0.2 and + 0.2 for each subject *n.* For each subject, also, we sampled two vectors with 10,000 values each: the first, ***s***_n1_, is Gaussian distributed (mean = 0, *SD* = 1), whereas the second is set as

$$s_{n2}=\beta_{n}s_{n1}+\varepsilon_{n},$$

where ***ε***_n_ is also Gaussian‐distributed. The vectors ***s***_n1_ and ***s***_n2_ constitute the unobserved neural process ***S***. The correlation between ***s***_n1_ and ***s***_n2_ can be analytically computed from *β*_n_ as

$$c_{n}=\beta_{n}/{\left({\beta_{n}}^{2}+1\right)}^{1/2}$$

We set the value of the observed behavioral variable for each subject to be

$$\chi_{n}=c_{n}+0.5\eta_{n},$$

where *η*_n_ is Gaussian distributed (mean = 0, *SD* = 1). Now, to sample the observed data sets ***D***_n_ for each subject, we randomly sampled *T* = 100‐time points from ***S***_n_ (whose columns are ***s***_n1_ and ***s***_n2_) and added some Gaussian noise with mean = 0 and *SD* = *σ.* We did this *R* times per subject, obtaining one (100 by 2) noisy data set ***D***_n_ = [***d***_n1_, ***d***_n2_] each time. We then set the observed replication values to

$$Y_{\mathrm{nj}}=z-\text{transformation}\;\left(\text{corr}\left(d_{n1},d_{n2}\right)\right),$$

where we applied the *z*‐transformation on the resulting correlation to make appropriate for parametric testing.

Note that, as illustrated in Figure 2, the (unobserved) value *β*n is involved in both the generation of ***Y***_n_ and ***x***_n_. With both ***Y***_n_ and ***x***_n_ in hand, we ran the described permutation testing algorithm on the noisily estimated FC matrix ***Y***_n_ and the behavioral variable ***x***_n_. By controlling *σ* (which defines how noisy are individual time series samples ***d***_n1_ and ***d***_n2_), we could make the detection more or less difficult.

We used a range of 30 values for *σ* between 0.25 and 1.5, and repeated the experiment, that is, data generation and testing, 100 times per value of *σ.* For each repetition of the experiment, standard permutation testing resulted on *R* = 100 *p* values (one per replication). Since *p* = 1, there was no need to control for familywise error rate across observed variables (Equation (3)).

**Figure 3:**
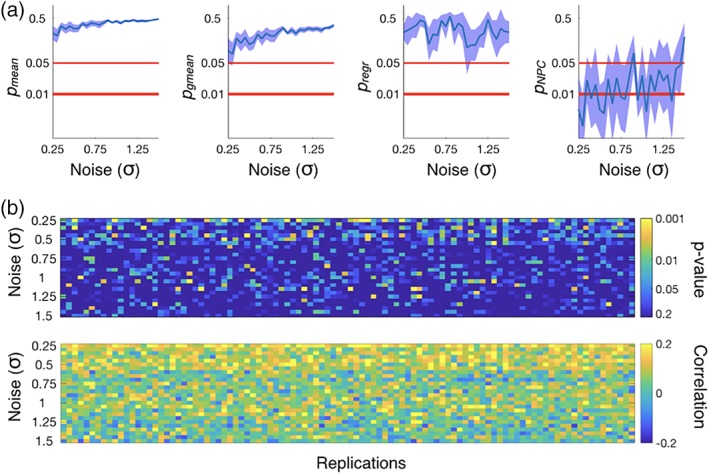
Results from the simulated data with *N* = 200, where there is a relationship between the tested variables: FC and behavior. (a) *p* Values obtained from combining tests using the mean (*p*_mean_ and *p*_gmean_), *p* values from the regression‐based permutation testing approach (*p*_regr_), and *p* values from the described permutation testing approach (*p*_NPC_), as a function of *σ*, which controls the noise in the replications (i.e., higher values of *σ* produce more difficult instantiations of the problem); 95% confidence intervals are computed across realizations of the experiment. (b) *p* Values before test combination for a given repetition (per value of *σ*), together with the estimated correlation coefficients [Color figure can be viewed at http://wileyonlinelibrary.com]

Alongside the NPC, we also ran for comparison the regression‐based permutation testing approach described above, denoted as *p*_regr_. Figure 3a shows *p*_mean_/*p*_gmean_/*p*_regr_/*p*_NPC_ (respectively from left to right) averaged across the 100 realizations of the experiment as a function of *σ*, together with 95% confidence intervals (minus/plus twice the standard error). We ran 10,000 permutations in each case. Thanks to the effect of the logarithm, the *p*_gmean_ values are lower than *p*_mean_ values, but neither of them ever reached significance provided the weak and volatile relationship between ***Y*** and ***x***. The individual per replication *p* values (shown in Figure 3b for one example repetition, per value of *σ*, together with the corresponding correlation coefficients) illustrate this point: although there were some significant *p* values, the average is condemned to fail due to the frequent bad *p* values associated to some too noisy replications. The *p*_regr_ values did not reach significance either, probably because of a loss of statistical power due to overfitting in the regressions (given that *N* = 200 is not much higher than *R* = 100). However, most of the *p* values from the NPC permutation approach turned out to be significant despite the low magnitude of the signal across replications, with the average of *p*_NPC_ across realisations of the experiment leaving the zone of significance only for the highest values of *σ* (i.e., for the hardest instantiations of the problem).

Supporting Information Figures S1 and S2 show additional simulations for *N* = 50 and *N* = 1,000 subjects, respectively. In the most difficult case, *N* = 50, both *p*_mean_ and *p*_gmean_ were far from any level of statistical significance, and *p*_NPC_, although exhibiting lower *p* values than *p*_mean_ and *p*_gmean_, reached significance only occasionally (but more often than *p*_regr_). In the easiest *N* = 1,000 cases, *p*_mean_ was under 0.05 for the lowest levels of noise, and *p*_gmean_ reached values under 0.05 for half of the range of *σ*; *p*_NPC_, however, stayed most of the time at the minimum levels allowed by the number of permutations (i.e., 1/10,001), clearly outperforming *p*_mean_ and *p*_gmean_. Comparatively, *p*_regr_ also reached significance for the entire range, but less strongly than *p*_NPC_. As observed, the NPC outperformed this alternative in every case. This was expected because univariate calculations are more robust to overfitting that multivariate regression, which hinders the latter's statistical power.

**Figure 4:**
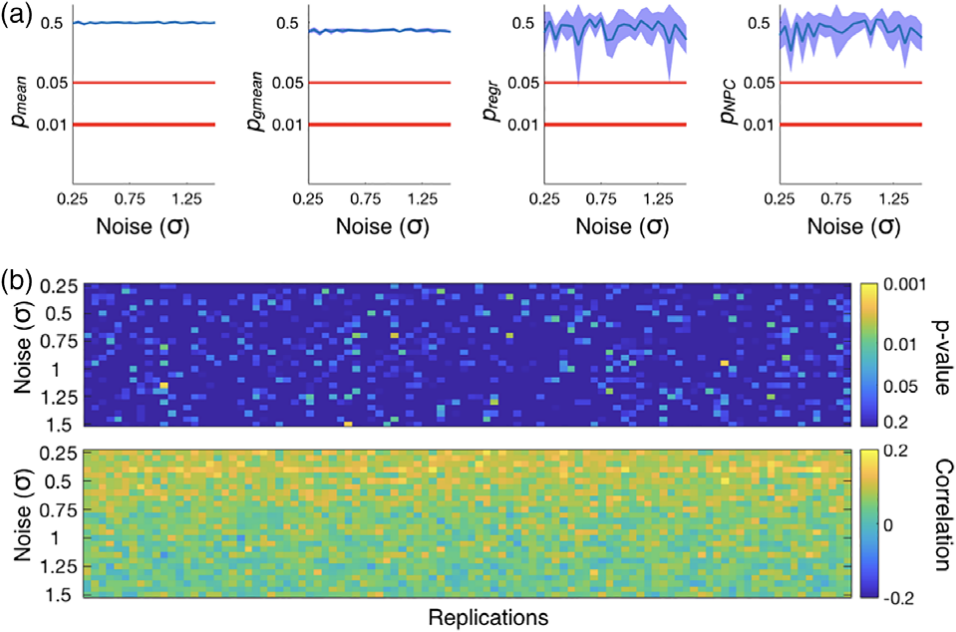
Results from the simulated data, where there is *not* a relationship between FC and behavior. The description of the panels is equivalent to Figure 3. In this case, however, the 95% confidence intervals do not overlap with the region of statistical significance no relation was found between FC and behavior, that is, there was no Type I errors [Color figure can be viewed at http://wileyonlinelibrary.com]

Next, we repeated the same analysis but forcing a fixed value of *β*_n_ for all subjects (in particular, we set *β*_n_ = 0). In this case, there is not a relationship between behavior and FC. Figure 4 shows that NPC, as well as the other methods, is robust to Type I errors.

## 4 DYNAMIC FUNCTIONAL CONNECTIVITY IN REAL DATA

Having demonstrated the utility of the NPC approach to relate FC to behavior in a synthetic scenario where the estimation was very noisy, we next evaluated it using real data by applying the Hidden Markov model (HMM) to resting state fMRI data from the Human Connectome Project (HCP). The HMM assumes that the data can be described using a finite number of states. Each state is represented using a probability distribution, which in this case is chosen to be a Gaussian distribution (Vidaurre, Smith, & Woolrich, 2017a); that is, each state is described by a characteristic pattern of BOLD activation and a certain FC profile (we use the same configuration as in Vidaurre, Smith, and Woolrich (2017a), to which we refer for further details). As the HMM is applied at the group level, the estimated states are shared across subjects; however, the state time courses that indicate the moments in time when each state is active are unique to a given individual. For the purposes of this analyses, we set the HMM to have 12 states. Note that, as discussed in former work (Vidaurre et al., 2018), there is no specific biological significance in the chosen number of states, and a different number of states just provide different levels of detail in the HMM decomposition. Here, we chose 12 states simply to be consistent with our previous work on this data set (Vidaurre, Smith, & Woolrich, 2017a). Using the inferred state time courses, the amount of *state‐switching* for each subject was calculated, which corresponds to a metric of how frequently subjects transition between different brain states (more specifically, given that the state time courses are probabilistic assignments, we compute the mean derivative of the state time courses for each subject). We used state‐switching as a summary metric of dynamic functional connectivity (DFC).

In order to infer the HMM at reasonable cost in spite of the large amount of data (820 subjects by four sessions by 15 min, TR = 0.75 s), we used a stochastic learning procedure (Vidaurre et al., 2017b), which involved performing noisy, yet economical, updates during the inference. Since stochastic inference brings an additional layer of randomness into the HMM estimation but is not costly to run, we repeated the HMM inference 100 times and computed state‐switching for each run. In this context, each HMM estimation constitutes a replication. Following the paper notation, we denote the state‐switching measure for subject *n* and replication *j* (averaged across the four sessions) as *Y*_nj_.

Although stochastic inference adds additional randomness to the estimation, the HMM has have previously been reported to perform very robustly in this data set (Vidaurre, Smith, & Woolrich, 2017a), possibly as a consequence of the large number of subjects (*N* = 820), the length of the scanning sessions, and the general high quality of the data. For this reason, the different HMM runs were quite consistent, which in turn means that the tests produce relatively similar results across replications (as shown below). To illustrate the effect of greater noise, we created a second set of replications where we permuted the state‐switching measure between subjects randomly for half of the HMM runs (i.e., half of the HMM runs, or replications, are potentially related to behavior whereas the other half are noise, and all of them are included in the analysis). We refer to this as the *perturbed* data, as opposed to the *original* data where the HMM estimations are left intact.

Furthermore, each subject has a number of behavioral measures, including psychological and sociological factors and several health‐related markers. We used a total of 228 behavioral variables, after discarding those with more than 25% of missing values, to test against DFC as measure by state‐switching. We included age, sex, motion, and body‐mass‐index (the latter two usually considered as confounds). We also discarded those subjects without family information and those with a missing value in any of the behavioral variables. We denote the (*N* by *P*) matrix of subject traits as ***X***.

We tested for significance in the correlation between switching rates across replications (***Y***) and each of the subject traits, contained in the columns of ***X,*** for both the original and the perturbed data set. We used 10,000 permutations, respecting the family structure of the HCP subjects (Winkler, Webster, Vidaurre, Nichols, & Smith, 2015).

**Figure 5:**
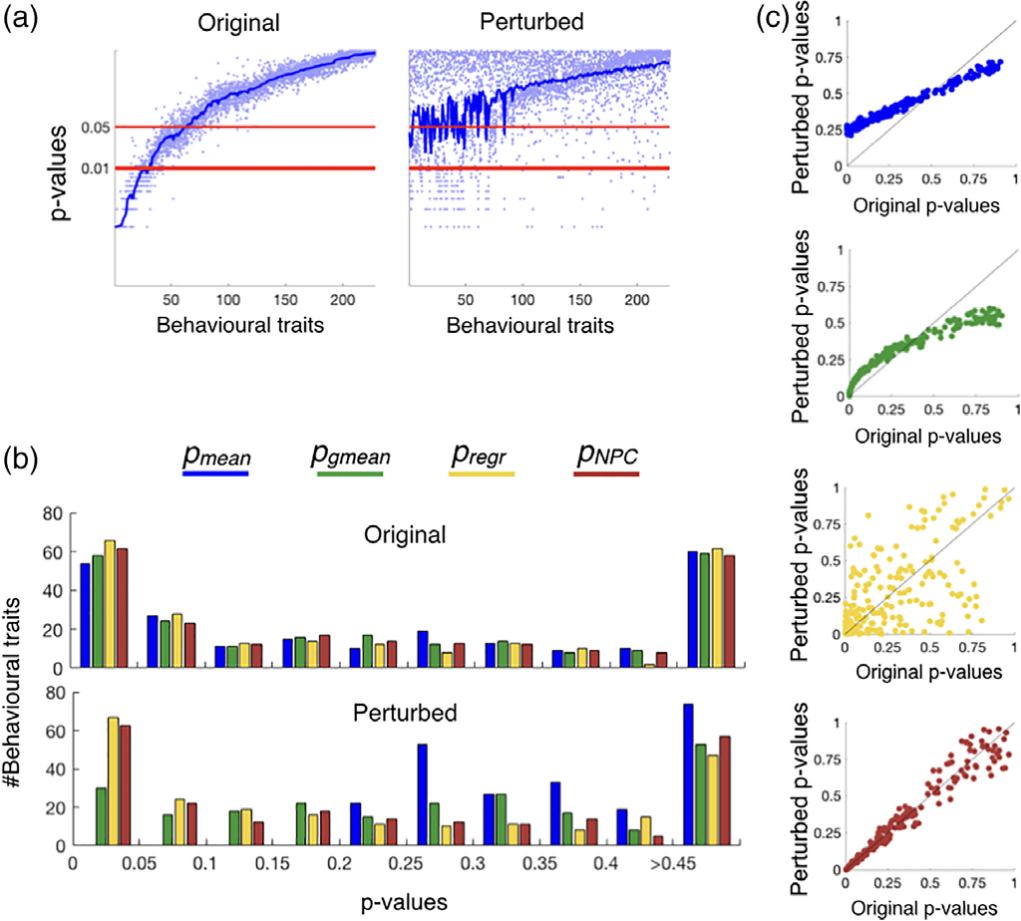
Analysis of the relation between behavior and DFC (state switching) as measured by the HMM, where replications correspond to HMM runs. (a) Mean *p* values (averaged over replications, with dots representing *p* values for a randomly chosen subset of 10% of the individual replications), reflecting the subject‐wise correlation of DFC with the different behavioral variables. On the *X*‐axis, behavioral variables are ordered from more to less correlated. On the left, this is shown for the original data set; on the right, this is shown for the perturbed data set (a noisier version of the original data set). (b) Histograms of *p* values, indicating that *p*_NPC_ and *p*_regr_ generally outperform *p*_mean_ and *p*_gmean_. (c) The *p* values are robust to perturbation only for NPC, where the correlation between perturbed and original *p* values is close to 1.0

Figure 5 compares the results of applying the NPC approach described above with the mean and geometric mean of the *p* values (*p*_mean_ and *p*_gmean_) as well as with the alternative regression‐based permutation testing outlined above (*p*_regr_). Figure 5a shows the mean *p* value (averaged across replications) reflecting the subject‐wise correlation of state‐switching (as measured by the HMM) with the different behavioral variables, with the behavioral variables being ordered from more to less significant; for purposes of illustration, dots represent individual *p* values for some randomly chosen replications. On the left, the *p* values obtained from standard permutation testing on the original HMM runs are quite consistent across replications; on the right, for the perturbed set of HMM runs, given that half were randomly ordered over subjects, the mean *p* value reflects the reduced effect strength.

In Figure 5b, we examine the histograms of *p* values for each of the four alternatives (with a loose use of the term “*p* value” when referring to *p*_mean_ and *p*_gmean_). On top, where all the HMM runs were used normally, the difference between methods is somewhat subtle. At the bottom, no variable was under significance level for *p*_mean_, and only 30 variables were under significance level for *p*_gmean_; in contrast, over 60 variables turned out to be significant for *p*_regr_ and *p*_NPC_. The difference between *p*_mean_ and *p*_gmean_ conveys the benefits of working on logarithm space, whereas the difference between *p*_gmean_ and *p*_NPC_ reflects the transformation needed to convert *p*_gmean_ to quantities interpretable as conventional *p* values. According to the small differences between *p*_regr_ and *p*_NPC_, the latter factor seemed to make the biggest difference in this data set. Regarding the regression‐based permutation method (*p*_regr_), given that we have 100 replications in this case and a large number of good‐quality subjects, the regressions did not suffer from overfitting as much as in the simulations above.

Figure 5c shows, for each of the methods, the (combined across replications) *p* values for the original data versus the perturbed data, reflecting that only the NPC approach was robust to having corrupted replications (i.e., the *p* values are almost identical between the original and the perturbed data set).

**Figure 6:**
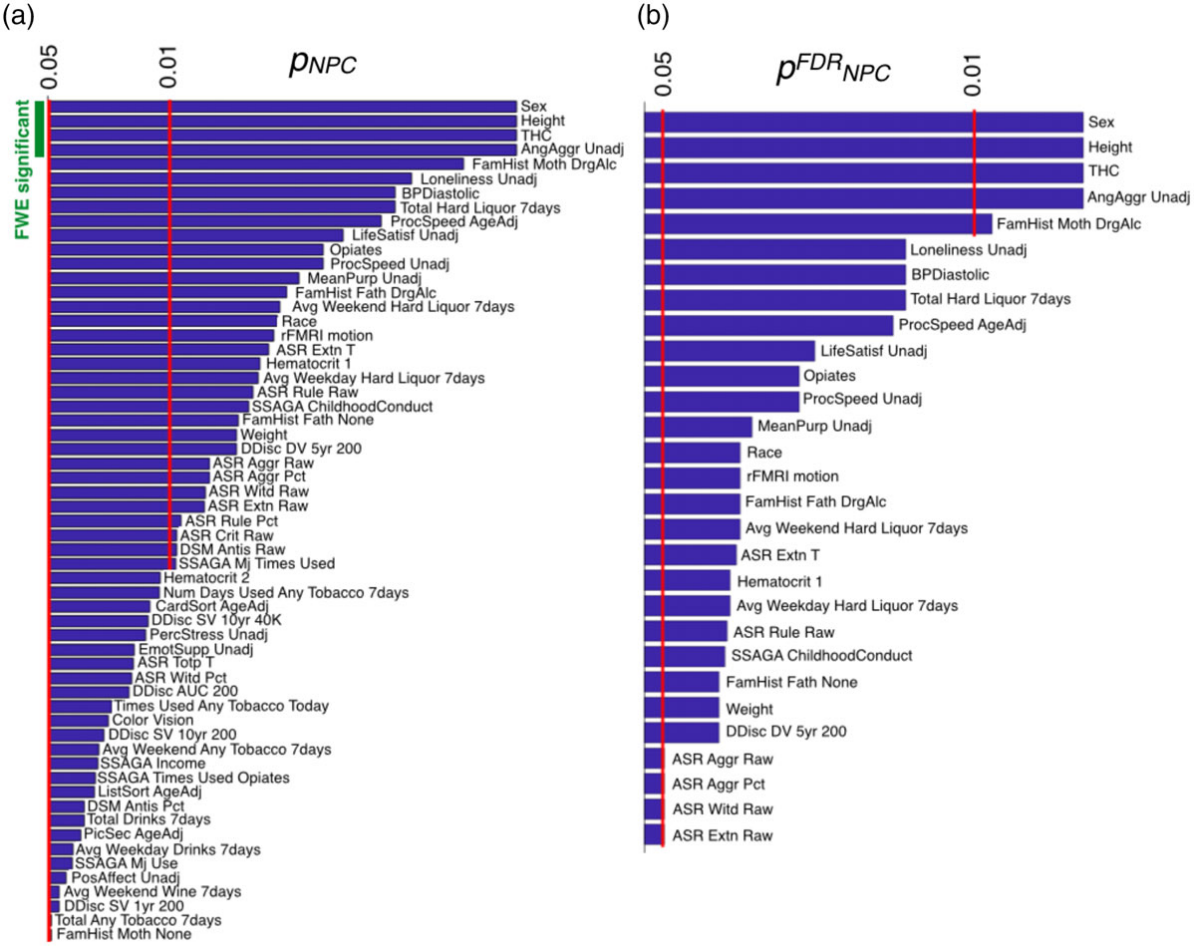
For the observed variables considered to be significant (out of 228), (a) *p* values using the NPC on *p*_gmean_ approach (*p*_NPC_), with FWE significance indicated on the top left; and (b) FDR‐corrected *p* values (*p* ^FDR^_NPC_) [Color figure can be viewed at http://wileyonlinelibrary.com]

Figure 6 presents the behavioral variables for which we found significance using the NPC procedure. Interestingly, although motion is a significant predictor it does not explain the greatest variance in this analysis, suggesting that DFC on resting state fMRI, as estimated by HMM, can be meaningfully related to behavior beyond the influence of motion. Most of the traits that were found significant were health‐related, with fewer higher‐level psychological traits than were found by Smith et al. (2015), which focused on functional connectivity instead of any dynamic aspects of the data (such as the state‐switching rate). Due to the relatively large number of observed variables, only a few were found to be significant after FWE correction (i.e., in Equation (3), the minimum of the surrogate *p* values across observed variables can be small if there are many observed variables to choose from). In contrast, FDR (Nichols & Hayasaka, 2003), allowed the identification of up to 25 variables. When we randomly corrupted the entire data set (instead of half of the subjects as in the perturbed data set), all methods, including NPC, were able to satisfactorily control for Type I errors (data not shown).

## 5 DISCUSSION

In this article, we show that the stochastic nature of FC estimations often considered a hindrance, can be effectively integrated to provide valid and sensitive inferential procedures. If the differences between the estimations are not only due to random noise but contain different elements of information, such integration can be largely beneficial. If these differences are just pure noise, the presented procedure can approximate the accuracy of a single, noise‐free estimation.

On these grounds, we describe a permutation testing approach based on previous work (Pesarin & Salmaso, 2010; Winkler et al., 2016) that can be used to test for the relationship between a set of observed variables and an unobserved (FC‐based) variable for which we have a number of noisy estimations. The crucial point is that we are not interested in finding the relationships as described by a particular FC estimation, but instead would like to understand the relationship of the *true* FC with the observed variables. We took as a concrete example the relationship between covert patterns of intrinsic brain connectivity, as they occur at rest, and patterns of cognitive and demographic variables measured outside of the scanner, using data from the Human Connectome Project.

Although we focused on univariate observed variables and replications, the described method can straightforwardly be extended in a number of ways. First, although we focused on linear relationships between variables, it can easily be extended to multivariate statistics, such as multivariate linear regression, or canonical correlation analysis. This is important in that it allows studies in which the mapping between cognitive function and the data is not univariate in nature. It can also be extended to situations when we have replications on both sides of the correlation, such as when both the observed and nonobserved behaviors are measured on multiple occasions. In this case, each pair of replications could be tested individually (for each element of the corresponding Cartesian product), and we would proceed similarly.

Moving forward, these types of approaches are likely to be particularly important in the domain of neuroscience given recent shifts toward the use of intrinsic connectivity at rest as a method of evaluating structural features of cognition. Intrinsic connectivity, as measured at rest, is a powerful tool for exploring the structure of neural organization since it is able to reveal similar patterns of neural organization as emerge during tasks (Smith et al., 2009). In addition, the simple noninvasive nature of the use of resting state as a method for assessing neural function means that it can be applied to multiple different populations, even those for whom task‐based measures of neural function or psychological measurements may be problematic (such as children or populations with cognitive problems). Measuring neural organization at rest is also easy to implement across centers making it amenable to the creation of large multicentre data sets, a shift that is likely to be increasingly important as neuroscience faces up to the challenges of reproducible science.

Despite the promise that assessing neural function at rest holds, many of the same features that make it an appealing tool for the cognitive neuroscience community are also at the heart of many of its limitations. For example, the power that is gained by the unobtrusive nature of the measure of neural function at rest also leads to concerns regarding what the measures actually represent: it is unclear which aspects of the neural signal reflect the intrinsic organisation of neural function, which reflect artefacts that emerge from physiological noise or motion (Power et al., 2012), and which reflect the patterns of ongoing experience that frequently emerge when individuals are not occupied by a demanding external task (Gorgolewski et al., 2014; Vatansever et al., 2017). In this context, because the underlying ground truth is unknown, an effective way to integrate estimations will help the researcher to identify which aspects of a given neural pattern are expressed in a robust way in relation to neurocognitive function.

Although dynamic approaches to understanding functional connectivity space are growing in popularity (Chang & Clover, 2010; Vidaurre, Smith, & Woolrich, 2017a), different approaches have specific limitations. For example, sliding window approaches depend upon an apriori selection of the window length, which limits the granularity of neurocognitive states that can be identified. While approaches such as HMM circumvent this problem by allowing the data to determine the temporal duration of the underlying states, these analyses are inherently probabilistic and parameter inference can introduce noise into the analysis. In this context, NPC allows dynamic approaches to cognition to be compared to observed data in a systematic manner. This could help pave the way to formally evaluate how different descriptions of the underlying dynamics at rest best predict variables with well‐described links to cognitive function. This way, NPC can become a useful tool in resolving the state–trait dichotomy that currently hinders the development of the science of how neural function evolves at rest.

## Supporting information


**Figure S1** Results from the simulated data, for *N* = 50 subjects. The description is as in Figure 3.

**Figure S2** Results from the simulated data, for *N* = 1,000 subjects. The description is as in Figure 3.

## Footnotes

1
https://github.com/vidaurre/HBM2018/blob/master/README.md.
