# Supplementary figures and images for: Stable between‐subject statistical inference from unstable within‐subject functional connectivity estimates
Source: Hum Brain Mapp. 2018 Oct 25;40(4):1234–43. doi: 10.1002/hbm.24442 (PMC6492297; doi:10.1002/hbm.24442)

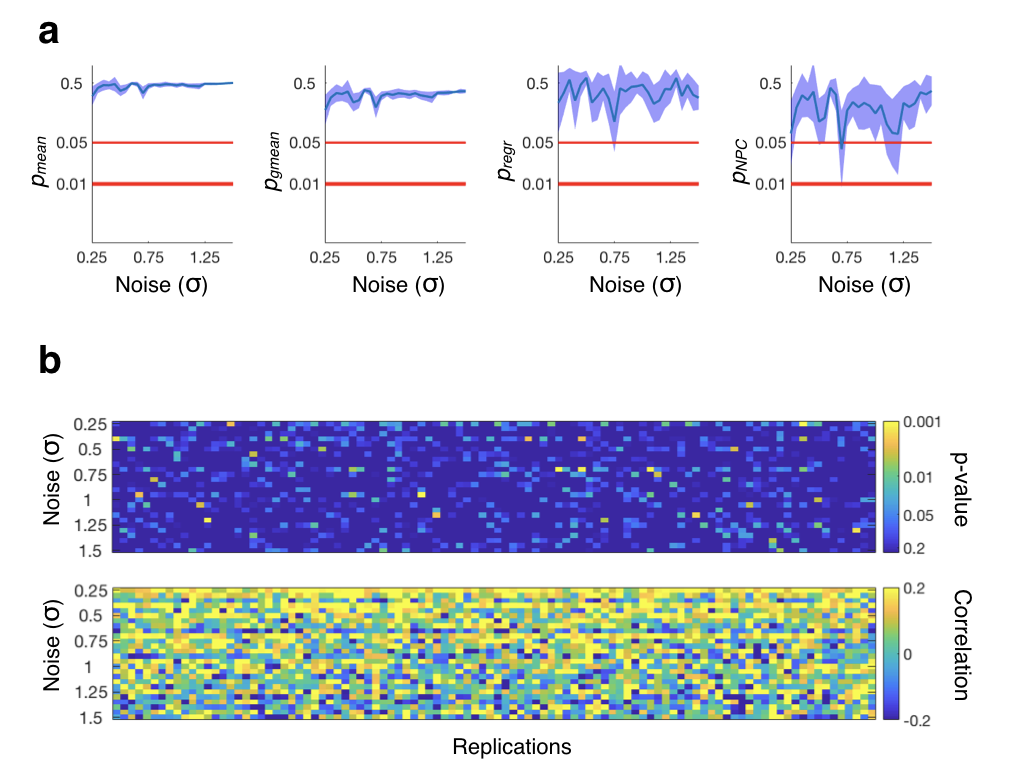

Supplement: Supplementary file 1 — Figure S1 Results from the simulated data, for N = 50 subjects. The description is as in Figure 3. [file HBM-40-1234-s001.tiff]

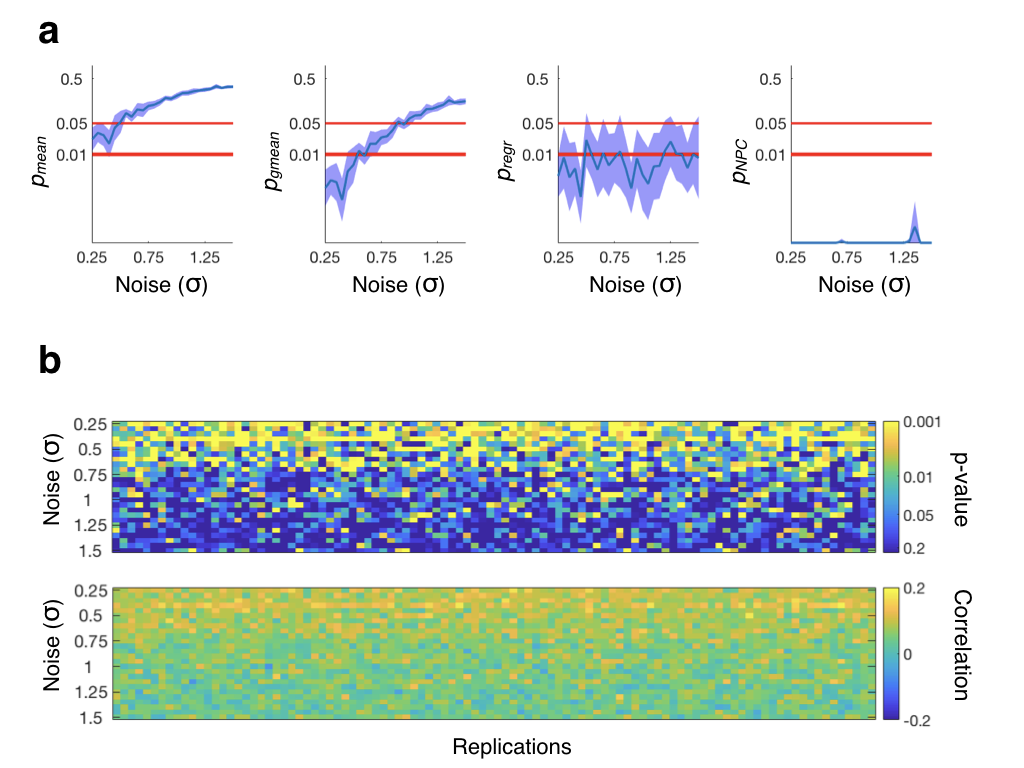

Supplement: Supplementary file 2 — Figure S2 Results from the simulated data, for N = 1,000 subjects. The description is as in Figure 3. [file HBM-40-1234-s002.tiff]
